# User’s Self-Prediction of Performance in Motor Imagery Brain–Computer Interface

**DOI:** 10.3389/fnhum.2018.00059

**Published:** 2018-02-15

**Authors:** Minkyu Ahn, Hohyun Cho, Sangtae Ahn, Sung C. Jun

**Affiliations:** ^1^School of Computer Science and Electrical Engineering, Handong Global University, Pohang, South Korea; ^2^Wadsworth Center, New York State Department of Health, Albany, NY, United States; ^3^Department of Psychiatry, University of North Carolina at Chapel Hill, Chapel Hill, NC, United States; ^4^School of Electrical Engineering and Computer Science, Gwangju Institute of Science and Technology, Gwangju, South Korea

**Keywords:** BCI-illiteracy, performance variation, prediction, motor imagery, BCI

## Abstract

Performance variation is a critical issue in motor imagery brain–computer interface (MI-BCI), and various neurophysiological, psychological, and anatomical correlates have been reported in the literature. Although the main aim of such studies is to predict MI-BCI performance for the prescreening of poor performers, studies which focus on the user’s sense of the motor imagery process and directly estimate MI-BCI performance through the user’s self-prediction are lacking. In this study, we first test each user’s self-prediction idea regarding motor imagery experimental datasets. Fifty-two subjects participated in a classical, two-class motor imagery experiment and were asked to evaluate their easiness with motor imagery and to predict their own MI-BCI performance. During the motor imagery experiment, an electroencephalogram (EEG) was recorded; however, no feedback on motor imagery was given to subjects. From EEG recordings, the offline classification accuracy was estimated and compared with several questionnaire scores of subjects, as well as with each subject’s self-prediction of MI-BCI performance. The subjects’ performance predictions during motor imagery task showed a high positive correlation (*r* = 0.64, *p* < 0.01). Interestingly, it was observed that the self-prediction became more accurate as the subjects conducted more motor imagery tasks in the Correlation coefficient (pre-task to 2nd run: *r* = 0.02 to *r* = 0.54, *p* < 0.01) and root mean square error (pre-task to 3rd run: 17.7% to 10%, *p* < 0.01). We demonstrated that subjects may accurately predict their MI-BCI performance even without feedback information. This implies that the human brain is an active learning system and, by self-experiencing the endogenous motor imagery process, it can sense and adopt the quality of the process. Thus, it is believed that users may be able to predict MI-BCI performance and results may contribute to a better understanding of low performance and advancing BCI.

## Introduction

Motor imagery based brain–computer interface (BCI) has become an increasingly prevalent process, and in recent decades researchers have made valuable achievements and demonstrated the feasibility of BCI for various applications, such as communication, control, rehabilitation, entertainment, and others (Wolpaw et al., 2002; Millán et al., 2010; Ortner et al., 2012; LaFleur et al., 2013; Ahn M. et al., 2014; Bundy et al., 2017; Guger et al., 2017). Despite such advances in BCI, there is still a significant hurdle to overcome before BCI can move forward to the public market. Reportedly, about 10 — 30% of BCI users do not modulate the classifiable brain signals that are critical to run a BCI system; such a phenomenon is called “BCI-illiteracy” (Blankertz et al., 2010). In recent years, researchers have investigated the BCI-illiteracy phenomena and proposed techniques to improve current BCI systems in the context of control paradigm, system feedback modality, algorithms, and training protocols (Hwang et al., 2009; Grosse-Wentrup et al., 2011; Kübler et al., 2011; Fazli et al., 2012; Hammer et al., 2012; Maeder et al., 2012; Ahn S. et al., 2014; Suk et al., 2014; Jeunet et al., 2015a, 2016a,c; Pacheco et al., 2017).

According to a recent review (Ahn and Jun, 2015), researchers have approached this problem from different perspectives, all aiming to answer one question: “What is the best correlate of performance variation?” The aims of such studies can be summarized as follows: First, identify what distinct characteristics exist in poor performers; second, understand why these traits are common in the lower performers; and, lastly, use the correlates to classify the poor performers in advance, prior to using BCI.

To resolve performance variation, many ideas have been proposed. These methods include introducing advanced signal processing techniques such as co-adaptive learning (Vidaurre et al., 2011; Xia et al., 2012; Merel et al., 2015), training users until they are able to generate classifiable signals (Mahmoudi and Erfanian, 2006; Hwang et al., 2009; Tan et al., 2014), and brain tuning that shifts the current brain state to a better state for motor imagery using tactile (Ahn S. et al., 2014) or electrical (Pichiorri et al., 2011; Wei et al., 2013; Yi et al., 2017) stimulation. These ideas are based on the fact that the human brain is able to reflect on, learn from, and even adapt to its experiences. In fact, the classical signal processing methods overlooked this aspect and thus focused on designing a better feature extractor or classifier than a fixed decoder (McFarland et al., 1997; Ramoser et al., 2000; Lemm et al., 2005). In recent years, advanced BCI system designs have been proposed to employ the concept of co-adaptation, which is the process whereby a user learns to control the BCI in conjunction with the adaptation of learning brain states of the user. However, in the studies of performance variation, such functionality of the brain is not seriously considered.

In general, the feedback modality of BCI system is considered important (Kübler et al., 2001; Hinterberger et al., 2004; Blankertz et al., 2006; Cincotti et al., 2007; Leeb et al., 2007; Nijboer et al., 2008; Gomez-Rodriguez et al., 2011; Ramos-Murguialday et al., 2012; McCreadie et al., 2014; Jeunet et al., 2015b, 2016b). Similarly, user feedback may be equally as important as system feedback. Interestingly, a few studies have evaluated feedback from users (Burde and Blankertz, 2006; Hammer et al., 2012; Vuckovic and Osuagwu, 2013). These studies used indirect measures to correlate with BCI performance. However, the variation of such parameters was not investigated while subjects performed the motor imagery tasks. Our ultimate aim is to predict a user’s performance prior to using the BCI system; therefore, it may make more sense to let users predict their performance directly. The brain can adapt to the external environment. Such functionality has been introduced in a co-adaptive learning algorithm in BCI systems; likewise, we expect that users may be able to directly estimate their own BCI performance. This idea is supported by a recent study by Vuckovic and Osuagwu (2013) that investigated the motor imagery quality indicated by users. The authors demonstrated that the proprioceptive sensation of a movement (kinesthetic imagery), which was assessed with 5-point rating scales, can be used as a test to differentiate between “good” and “poor” performers in motor imagery BCI.

Several benefits are conceivable with the self-prediction of BCI performance. First, it removes or reduces time-consuming preparation or installation and recording procedures that usually require tremendous effort from users and experimenters. Note that relevant studies employed brain signal recording (Blankertz et al., 2010; Ahn et al., 2013a,b), brain imaging (Halder et al., 2011, 2013) and intensive psychological tests (Hammer et al., 2012). However, if self-prediction is possible and usable, the only necessary step is to ask the user how his/her BCI performance will be based on his/her own feelings or experiences. This is the best scenario because it does not require any further behavioral, psychological, or experimental tasks. In a study by Vuckovic and Osuagwu (2013), subjects also conducted some behavioral and mental tasks, and they could therefore answer questions based on their sensations. Therefore, the best-case scenario, in which the user immediately answers questions about his/her performance, is less likely. Rather, the user may need to perform some tasks—either behavioral, mental, or motor imagery—to give enough experience and time to build the sense of their individual motor imagery proficiency. Even if this is proven true, however, we can still reap the other benefits of self-prediction. BCI performance may be somewhat influenced by unique characteristics of the user and the hardware/software of the system (Kübler et al., 2011). Therefore, a BCI user may realize that he/she is good or bad at conducting motor imagery at some point during the training or testing of BCI; the user is then able to clarify where or when the BCI problem occurs. This is the second benefit. The third benefit is that the inferior data can be separated from the good dataset. This is important because poor-quality data is much more likely to introduce a bias in classification results and can eventually lead to incorrect conclusions in research.

In summary, the brain is a good learning system and motor imagery is an endogenous process. Therefore, a user’s self-assessment provides valuable feedback and includes information relevant to understanding performance variations. However, to the best of our knowledge, this aspect has not been well investigated in the existing studies on BCI illiteracy; thus, a rigorous analysis on it would be quite interesting and informative. To address the issue of whether a user’s self-predicted score is correlated with offline BCI performance and, if so, how long it may take for the user to get a sense of the connection, we investigated the user’s self-assessed parameters, including mental and physical states, the quality of motor imagery, and self-predictions of motor imagery performance before and between runs.

## Materials and Methods

### Motor Imagery Task

In this study, 52 healthy subjects (26 males, 26 females; mean age: 24.8 ± 3.86 years) participated. Among all subjects, six subjects experienced BCI or biofeedback with neurophysiological signals. The Institutional Review Board from the Gwangju Institute of Science and Technology approved this experiment. All participants were informed of the purpose and process of the experiment and each written consent form was collected before conducting the experiment. We used BCI2000 software (Schalk et al., 2004) and Biosemi Active 2 system (64 channels, sampling rate: 512 Hz) for stimulus presentation and electroencephalography (EEG) signal acquisition, respectively. At the beginning of the experiment, we recorded 1-min resting state signals under eyes-open conditions, and we then conducted a conventional two-class motor imagery experiment. A detailed step-by-step description of the motor imagery experiment follows.

**FIGURE 1:**
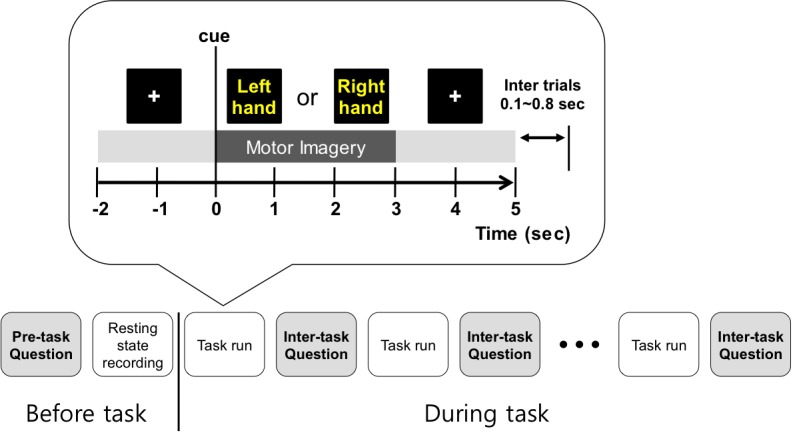
Overall experiment design. Before the 1st run, personal information was collected, and subjects practiced motor imagery and predicted their classification accuracy. Then, resting state EEG was recorded. Between tasks, the follow-up questions were asked to collect the self-assessed condition levels and motor imagery related scores, including prediction of BCI performance.

On the monitor screen, the visual instruction was presented. At the beginning of each trial, a cross appeared for 2 s, and then text indicating left or right was shown for 3 s. Subjects were asked to imagine left- or right-hand movement according to the presented direction at the motor imagery phase. Right after the motor imagery phase, a cross appeared for 2 s again. Thus, the total time of each trial was 7 s and the inter-trial interval was set randomly to between 0.1 and 0.8 s. In each run, 20 trials for each condition (left or right) were collected, and at the end of each run we asked the subject if he/she could perform the next run. Only when the subjects responded ‘Yes’ would they perform the next run. All subjects conducted 5 or 6 runs with about a 2-min break between runs. Thus, we obtained a total of 100 or 120 trials for each condition for each subject. During the experiment, there was no feedback given on how well subjects performed the motor imagery tasks. The procedure for one trial is illustrated in **Figure 1**.

### Questionnaire Survey

Prior to the motor imagery experiment, the subjects were asked to practice kinesthetic imagery rather than visual imagery since kinesthetic imagery yields better discriminable brain wave patterns than visual imagery does (Neuper et al., 2005; Vuckovic and Osuagwu, 2013). An explanation regarding the meaning of binary classification was also given, so subjects would understand the concept of poor (50%) and perfect (100%) accuracies. Thus, most of the subjects answered between 50 and 100%, with the exception of six subjects (sbj7, sbj21, sbj28, sbj36, sbj51, and sbj52) as seen in Supplementary Table 1.

Two questionnaires were generated: pre-task and inter-task forms (**Table 1**). Each subject filled out a pre-task questionnaire that contained question items regarding age, handedness, sex, hours the subject had slept the previous night, hours elapsed since the subject had ingested substances (coffee, alcohol, or cigarettes), and predicted accuracy. Then the subject started the actual motor imagery task. In order to obtain the feedback about physical, mental, and emotional states and motor imagery scores of each run, an inter-task questionnaire was provided at the end of each run, and the subject filled out the form.

**Table 1:** Question items for motor imagery experiment.

Before experiments	Personal information
(pre-task question)	• Handedness (R: right, L: left, and B: both hands)
	• Sex (M: male, F: female)
	• Age
	• Hours the subject slept the previous night (SLP)
	• Hours elapsed since the subject had coffee (COF), alcohol (ALC), or a cigarette (CIG)
	Motor imagery scores
	• Accuracy prediction (PreAP)

After each run	Self-assessed physical, mental and emotional states
(inter-task question)	• Calmness (CALM)
	• Interest (INT)
	• Concentration level (CONT)
	• Physical state (PHYS)
	• Mental state (MENTAL)
	• Fatigue (NSLP; number of times the subject fell asleep during the run)
	Motor imagery scores
	• Easiness of motor imagery (ES)
	• Accuracy prediction (AP)

Subjects scored the easiness of motor imagery on a scale from 1 to 5 (1: easiest to 5: very difficult) and the prediction of their classification accuracies between 50 and 100%. We note that the subjects were not given any feedback on their motor imagery performance during the experiment; their predicted motor imagery scores were purely based on their own experiences or expectations. In conducting the self-assessment of physical, mental, and emotional states (except for fatigue), each question was answered according to a 1–5 scale (1: most or best to 5: least or worst). The overall procedure of the experiment is illustrated in **Figure 1**. For detailed experiment information and questionnaire forms, refer to the literature (Cho et al., 2017).

### Motor Imagery Classification Accuracy

Motor imagery classification accuracy was calculated in a conventional way, by using Common Spatial Pattern (CSP) and Fisher Linear Discriminant Analysis (FLDA) (Ramoser et al., 2000; Ahn et al., 2013b). For the statistically reasonable accuracy, different groups of training and testing trials were generated multiple times (120 repetitions), and the final accuracy was obtained from this cross-validation method. The detailed procedure is as follows. First, EEG signals were filtered with the specific frequency band (8–30 Hz) and temporal interval (0.4–2.4 s after cue onset) to include the informative event-related (de) synchronization of Alpha/Beta (Pfurtscheller and Lopes da Silva, 1999; Ahn et al., 2012), which was reported as the primary feature in motor imagery BCI. With the filtered epochs, the cross-validation technique was applied to produce a statistically reasonable estimate of classification accuracy. Data was processed by first grouping all trials into 10 subsets of equal size; these 10 subsets were separated into seven training and three testing sub-groups. Therefore, the total number of such possible separations is 120. For each separation, the 10 most significant spatial filters were extracted from training trials by CSP and then a class separation line through FLDA was generated. By applying these 10 spatial filters and the classification line to corresponding testing trials, the correct rate (the number of correct epochs divided by the number of total epochs) was estimated. This procedure was repeated for other separations. Finally, all correct rates were averaged and this average was defined as the motor imagery classification accuracy.

### Analysis

In the analysis of personal information, for each comparison we divided subjects into two groups according to Sex (male vs. female) or Age (older vs. younger than 24.8 years, which is the mean age of subjects), or Coffee/Alcohol/Cigarettes (subjects who had coffee/alcohol/cigarettes within 24 h vs. subjects who did not) since the data showed that many subject answered that they did not consume the substances. Most of the subjects were right-handed, thus the handedness classification was excluded for further analysis. Then, the offline classification accuracy was compared at the in-group level. Here, the Wilcoxon Rank-Sum test was applied to check statistical significance. For the other questions, which had 5-scale scores, we first flipped the self-assessed scores in the following way: score 1 switched to score 5, 2 switched to 4, and so on. Then, the correlation coefficient between the scores (for each personal information category) and motor imagery classification accuracy over subjects and corresponding *p*-values were calculated using the MATLAB “corr()” function, which computes Pearson’s Correlation. For the statistically reasonable results, a permutation test was conducted (*n* = 2,500) and correction for multiple comparisons was made for *p*-values using the False Discovery Rate (FDR) (Benjamini and Hochberg, 1995; Genovese et al., 2002) with a *q* value of 0.1.

These processing steps made the results more intuitive; if results from one question item were highly correlated to the motor imagery classification, it would show an overall positive correlation. For pre-task questions, the raw answer values were used, but the scores in the inter-task questions were averaged over runs for further analysis. The Pearson’s correlation coefficient was also computed with actual classification accuracy. A permutation test in which actual BCI performance is shuffled was also conducted (*n* = 2,500) and corrected for the statistical significance.

For investigation of the variations of self-prediction over time, session-wise classification accuracy was compared with the self-prediction at each run. The correlation coefficient and the corresponding Root Mean Square Error (RMSE) were quantified. In addition, the cross-validated classification accuracy at each run was computed using the same method (six CSP filters and FLDA) and compared with the prediction to see the evolution of run-wise accuracy and prediction across runs. The result from this analysis will be used in the Section “Discussion.”

## Results

### Personal Information and Conditions

The scores from the questionnaire survey and offline classification accuracy are tabulated in **Table 2** (for accuracy prediction at each run, see Supplementary Table 1). The overall classification accuracy yielded 66.93 ± 11.05% (average ± standard deviation), ranging from a minimum of 46.9% to a maximum of 96.1%. We observed that the classification accuracies were well scattered, and our collected datasets therefore had a broad spectrum of subjects from poor to good performers in terms of motor imagery proficiency.

**Table 2:** Questionnaire results and neurophysiological index. For detailed information about items, please see the text.

ID	Hand	EXP	SLP	COFF	ALC	CIG	CALM	INT	CONCENT	BODY	MENTAL	NSLP	preAP	ES	AP	ACC	ACCSTD
sbj1	R		4	5	0	0	5	4	3	3	3	0.4	80	3.8	88	96.11	2.60
sbj2	R		3	0	0	0	4	3.8	3.8	3.4	3.4	0.3	50	4.4	74	95.65	4.46
sbj3	R	Yes	3	0	0	0	4.2	3.6	3.6	3.8	3.4	0	70	3.4	71	84.60	5.33
sbj4	R		2	20	15	0	4.2	3.6	3.6	4	4	0	70	4	75	83.07	4.46
sbj5	R		4	0	0	0	4	4	4.2	3.6	3.2	0.8	80	4.6	85	81.97	4.66
sbj6	R		3	0	22	0	3	2.6	3.6	2.8	3.4	0	65	3	68	79.65	4.47
sbj7	R		1	2	0	1	5	3.6	2.8	1	2	0.4	50	2.8	72	79.18	8.15
sbj8	R		1	2	0	1	4	3	3.6	4	3	2.4	75	3.6	69	78.96	6.94
sbj9	R		2	30	16	16	5	4.4	4	2	4	0	60	1.6	56.2	78.89	6.53
sbj10	R		3	0	0	0	4	2	2	3	3	0.7	70	2.4	64	78.72	6.73
sbj11	R		3	17	0	0	2.8	4.4	2.8	3.4	3.8	0	80	3	65	76.90	5.05
sbj12	R		3	16	0	0	4	4	4.4	4.8	4.4	0	80	4.2	70.6	76.00	8.20
sbj13	R		3	0	0	0	5	4.2	4.4	4.4	4.4	0.2	75	4.2	72	75.33	8.85
sbj14	R		1	23	0	0	4.2	1.8	4.4	3	3	0	80	3.2	65.6	75.19	5.03
sbj15	R		5	0	17.5	0	4	2.4	3.4	4	3.8	3.2	60	4	63.8	74.24	5.61
sbj16	R		4	22	0	0	2.4	2.2	2.2	3	2.8	1	60	3	62	73.64	6.05
sbj17	R		4	1	0	0	4.4	4.8	4.2	5	4.6	0.4	80	4	75	72.54	6.49
sbj18	R		2	12	0	0	3	4	3.6	3.2	4	0.6	80	3.8	72	71.44	6.10
sbj19	R		3	0	14	14	4	2	3	4	4	0	70	4	66	71.44	6.43
sbj20	R		2	0	0	0	4.8	3.6	3	3	3.4	0.4	50	2.4	58	70.99	5.50
sbj21	B		4	7	0	0	3	3	3	3	3	0	70	2.4	56	69.33	6.85
sbj22	R	Yes	4	0	0	0	5	4	5	5	5	0.4	90	5	86	68.33	6.30
sbj23	R		3	0	0	0	3.8	3.4	3.6	4	4	0.8	75	2.6	59	66.47	6.30
sbj24	R		4	4	0	0	5	3.6	3.6	4	4.2	0.4	75	4.2	65	66.42	6.38
sbj25	R		2	7	0	0	3.8	2.6	2.6	3.2	3.2	0	70	3.2	56	64.50	5.96
sbj26	R		3	0	8	0	3.8	3.2	3.2	3.2	3.2	1.2	80	3.8	60	63.63	6.27
sbj27	R		3	2	0	0	3	3	3	3	3	1.3	60	3	61	63.22	5.84
sbj28	R		2	0	0	0	4.2	2.8	3	4	3.8	0.6	90	2.6	52	63.14	6.27
sbj29	R		2	0	0	0	5	2.6	4	5	5	0	50	2	61	63.04	6.98
sbj30	R		4	0	0	0	4.6	3.2	4.2	4.2	4.4	0	60	4	58	62.96	5.97
sbj31	R		2	5	0	0	3	3	3	3	3	0	50	2	50	62.43	5.26
sbj32	R		3	0	0	2	4	4.2	3.4	3.8	4.2	0	65	3.4	61	61.17	5.19
sbj33	R		4	0	0	0	3.2	2.6	2.8	2.8	3	0	70	2.2	62	61.13	5.15
sbj34	R		2	0	0	0	5	4.8	3.4	5	5	0	75	3.2	60	60.83	5.28
sbj35	R		3	0	0	0	3.8	3.6	3.6	5	5	0	65	4	58	60.35	5.95
sbj36	R	Yes	3	0	0	0	3	2.8	2.4	2.4	2.8	1	60	2.2	61.25	60.18	4.87
sbj37	R		4	12	0	0	3.6	4.2	3.8	3.4	3.6	0	70	4	59	59.71	6.69
sbj38	R		2	0	0	0	3.8	3.4	2.2	3	2.8	0	70	1.6	52	59.19	5.31
sbj39	R		4	0	0	0	4	3	2.6	2.8	2.6	0.6	75	2.8	61	58.07	6.42
sbj40	R		5	0	0	0	4.4	3.6	4	4	4	0	81	2	50.4	57.69	5.54
sbj41	R		5	0	0	0	3	2.6	2.2	4.8	4.8	0	80	1.8	52	57.64	5.71
sbj42	R	Yes	3	0	0	0	4	4	3.2	3.8	3.6	0.4	80	3	62	57.29	6.54
sbj43	R		2	0	23	0	3.8	3.2	3.2	4	4	0.6	70	2.8	59	56.76	5.37
sbj44	R		1	2	0	0	3	3	2.6	3	2.6	0.3	60	2.8	56	56.68	5.54
sbj45	B		2	4	0	0	3	3	3	3	3	0.2	70	3	64.2	55.81	5.53
sbj46	R	Yes	3	0	0	0	4.8	4.8	4	4	4	0	80	3.6	58	55.40	6.01
sbj47	R	Yes	5	0	0	0	3.4	1.2	1.8	2.6	2.6	0	80	2.6	64	54.57	5.01
sbj48	R		4	0	0	0	3.4	2.4	2.8	3.2	2.6	4.8	83	4.8	66	54.11	6.48
sbj49	R		1	11	0	0	3.8	4	3.2	2.8	2	0	80	3.4	62	53.16	5.31
sbj50	R		4	6	0	0	4	4	3.6	3.6	3.6	0	70	2.6	58	52.58	6.11
sbj51	R		4	0	0	0	4	4	2.8	3.8	3.8	0.8	0	2.4	55	52.14	5.46
sbj52	R		2	0	0	0	3.4	3.4	3.4	3.2	3.4	1	70	3	66.25	47.90	6.03

**FIGURE 2:**
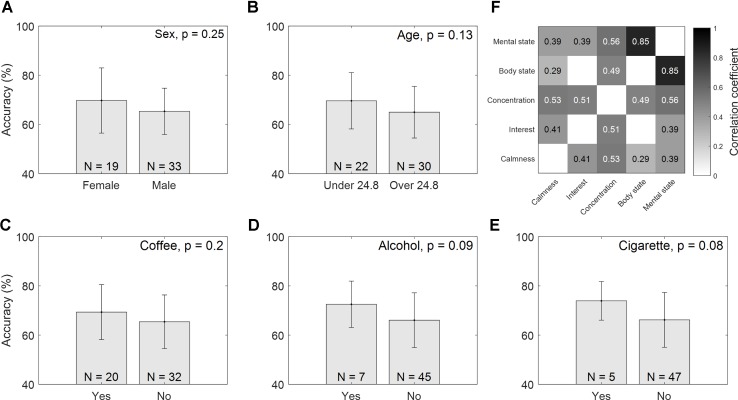
Accuracy comparisons between different groups. Mean accuracy with standard deviations are presented, and the total number of subjects within each group is noted on the bottom of each figure: **(A)** Sex, **(B)** Age, **(C)** Coffee, **(D)** Alcohol, and **(E)** Cigarettes. Figure **(F)** notes the correlation coefficients between physical/mental scores (FDR-corrected).

**Figures 2A–F** show the accuracy comparisons of different groups classified according to Sex, Age, Coffee, Alcohol, and Cigarettes. There are apparent differences between groups. Young female subjects seemed to perform better than male and relatively older subjects who were more than 24.8 years of age. Additionally, subjects who had coffee/alcohol/cigarettes in the 24 h preceding the tests were likely to show higher motor imagery accuracy than the subjects who had not. However, these differences were not statistically significant (Sex: *p* = 0.25, Age: *p* = 0.07, Coffee: *p* = 0.2, Alcohol: *p* = 0.09, and Cigarettes: *p* = 0.08). In the correlation analysis of physical and mental states, none of the items (SLP: *r* = -0.06, NSLP: *r* = -0.04, Calmness: *r* = 0.25, Interest: *r* = 0.08, Concentration: 0.25, Physical state: -0.08, or Mental state: *r* = 0.0 with *p* > 0.05 for all results) were significantly correlated with classification accuracy, although they were highly correlated to each other, as shown in **Figure 2F**.

### Easiness of Motor Imagery and Self-Prediction of Accuracy

**FIGURE 3:**
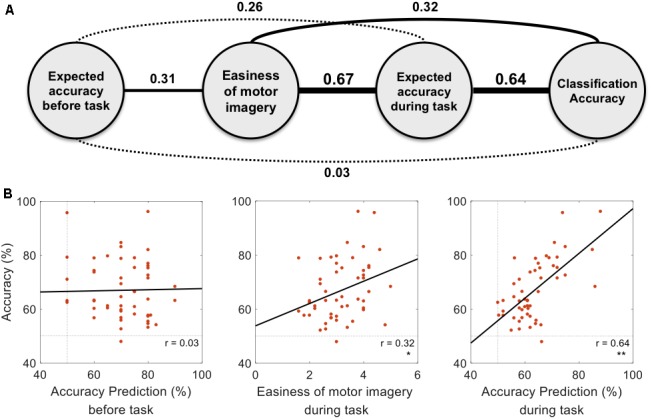
Correlations with actual classification accuracy. **(A)** The relationships between measures are presented with correlation coefficients. The non-significant correlation (*p* > 0.05) and significant correlation (*p* < 0.05) are presented with dotted and solid lines, respectively. Line width represents the strength of correlation. **(B)** Dots in each figure represent subjects, and the black line is the linear regression line to data points. Statistical significance is marked with one star (*p* < 0.05) or double stars (*p* < 0.01) on the right bottom of each figure (*p*-values were FDR corrected).

**FIGURE 4:**
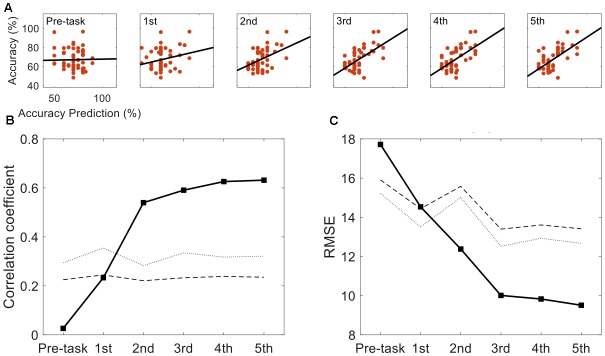
Self-prediction comparison across task runs. **(A)** The evolution of accuracy and self-prediction from pre-task to the 5th run. **(B)** Correlation coefficients between self-predicted performance and actual classification performance are presented across pre-task and the 1st to 5th runs. **(C)** Corresponding Root Mean Square Error between the predicted performance and actual classification performance are presented. Statistical lines are marked with the dotted (*p* = 0.01) and the dashed (*p* = 0.05) lines.

**Figure 3** represents the results of the correlation analysis. Most measures were positively correlated with classification accuracy, but the magnitude of this correlation (how strong) varied depending on the measures. The predicted accuracy during runs (the average of many predictions assessed during runs) showed a higher correlation coefficient (*r* = 0.64, *p* < 0.01, permutation test *n* = 2,500) than easiness of motor imagery (*r* = 0.32, *p* < 0.05, permutation test *n* = 2,500), even though they are quite positively correlated (*r* = 0.67). On the other hand, the classification accuracy which subjects predicted before the playing task yielded no significant correlation with offline classification accuracy (*r* = 0.03, *p* > 0.05, permutation test *n* = 2,500). It is inferred from these results that during the task, subjects could more accurately estimate their motor imagery proficiency to some extent. However, they were not able to do so before completing the task. A more in-depth analysis was conducted with the individual estimates at each run. **Figure 4A** demonstrates how self-prediction changes as the subjects participate in each task from pre-task to 5th run. The increasing tendency of the correlation coefficient was clearly observed, and it dramatically jumped from the pre-task expectation (*r* = 0.03) to the 1st (*r* = 0.23) and 2nd (*r* = 0.54) runs in **Figure 4B**; it then increased marginally afterward. Similarly, as shown in **Figure 4C**, RMSE steeply decreased from pre-task (RMSE = 17.7%) to the 3rd run (RMSE = 10%) and then fluctuated slightly. Permutation tests (*n* = 2,500) revealed that correlation coefficient and RMSE in the 2nd to 5th runs are statistically different from the two statistical ceilings [*p* = 0.05 (dashed line) and *p* = 0.01 (dotted line)].

## Discussion

In this study, we instructed subjects to predict their motor imagery performance and investigated how their predictions correlated with actual (offline) classification accuracy. Initially, participants estimated their performance poorly. As observed in the pre-task and 1st run in **Figure 4B**, the correlation coefficients were determined to be non-significant from the permutation test; however, as participants completed the motor imagery task (from 2nd to 5th runs, the correlation coefficients were statistically significant and increasing), they seemed to get a better sense of their motor imagery quality.

Subjects evaluated how easy their motor imagery was, and the self-assessed motor imagery scores wound up being positively correlated with offline classification accuracy. This relationship concurred with the results of the reports from Vuckovic and Osuagwu (2013) and Marchesotti et al. (2016), which stated that simply asking questions about the quality of motor imagery can be used to predict motor imagery performance. Interestingly, the predicted accuracy during tasks was also highly positively correlated with actual classification accuracy (*r* = 0.64, *p* < 0.01), and this value was notably larger than the correlation coefficient of scores on the easiness of motor imagery (*r* = 0.32, *p* < 0.05).

Considering Kübler et al.’s (2001) statement that “perception of the electrocortical changes is obviously related to the control of these changes,” perception is an important feature to understand the internal state, and the self-report is the tool to express such information to the external world. Related to this notion, the above-mentioned results raise an interesting question. The information that the subjects used to predict their performance accuracy may have come from the same source they used for evaluating the easiness of motor imagery, so both measures may overlap in a certain domain. From our results, it is believed that this is true, since the two measures are highly correlated (*r* = 0.67), as shown in **Figure 3A**. However, we observed a greater difference between the two measures in terms of the correlation coefficient with actual classification accuracy. Considering that many psychometrics by self-report (e.g., vividness of motor imagery, mental rotation, and visuo-motor coordination), were confirmed to be significant measures for predicting an individual’s BCI performance (Hammer et al., 2012; Jeunet et al., 2015a; Marchesotti et al., 2016), self-assessment seems to be a useful tool for evaluating motor imagery. These results demonstrate the psychological capacity of sensing the internal state of a participant based on a certain model that depends on the given question or task to perform.

In this study, subjects might develop the internal model of evaluating themselves in the accuracy domain. Easiness of motor imagery is simply a score selected from a 1 to 5 scale. However, to predict classification accuracy, subjects were given an explanation regarding the method of classification and the ranges of accuracy (50–100%). Thus, in transforming the subject’s actual feeling regarding the quality of motor imagery to the other domain—the accuracy measure—the self-evaluated value seems to become more like the form of accuracy. From this result, we learned the following important lessons. First, directly estimating the target measure (here, BCI performance) that researchers want to see may be better than correlating the indirect value (any correlates) with the target measure. Second, subjects should be given sufficient information on what they are evaluating.

Based on our results, the ideal case—wherein subjects predict their performance in a short time without performing any tasks—may not be achievable; they need time to develop an accurate sense of how to evaluate their motor imagery quality and to predict BCI performance. In this context, an interesting question is how many trials are sufficient for users to build a sense of predicting BCI performance. **Figure 4** gives us a brief clue. At the 1st and 2nd runs, the correlation between BCI performance and self-prediction showed dramatic increases, and the RMSE steeply decreased from 17.7% (pre-task) to 10% at the 3rd run. Because 40 motor imagery trials (for both left and right imagery) were collected at each run, it is inferred that 80–120 motor imagery trials (roughly 9–16 min in time) would be required for subjects to develop the ability to achieve relatively accurate self-assessment. In conclusion, BCI-illiterate persons could stop at about half the number of planned tasks (the planned number of trials: 200 or 240) in our experiment.

Related to the evolution of self-prediction, another interesting question is whether users can recall the experience from one session and accurately evaluate themselves in the following session. If that were the case, then it would be possible for participants to accurately predict their performance without having training time to sense their abilities. Multi-session data will help to answer that question.

**FIGURE 5:**
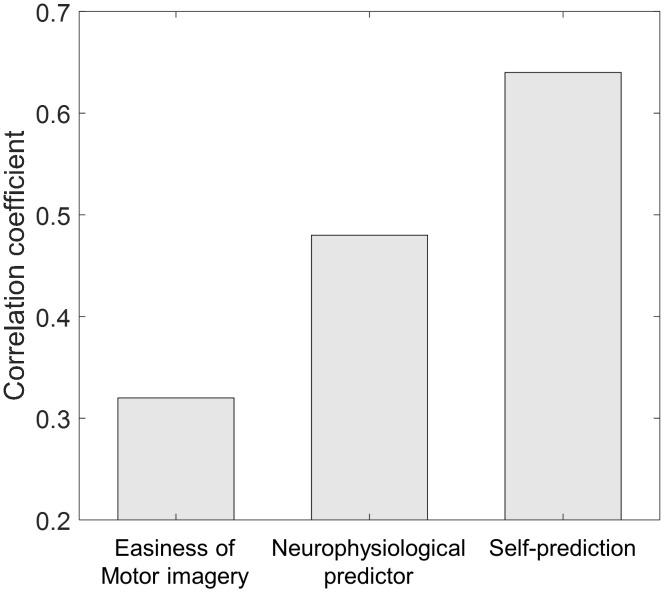
Correlation coefficient comparison across different methods. For the neurophysiological predictor, the value from the study by Ahn et al. (2013b) was adopted.

Another interesting question is whether self-prediction is better as a predictor for BCI performance than other correlates. In the literature, various types of correlates were investigated and researchers reported the following correlation coefficients: *r* = 0.59 (Burde and Blankertz, 2006) and *r* = 0.50 (Hammer et al., 2012) in psychological parameters; *r* = 0.72 and *r* = 0.63 (Halder et al., 2011, 2013) in anatomical parameters; and *r* = 0.53 (Blankertz et al., 2010) in neurophysiological parameters. Results from our previous study indicate that low alpha and high theta are the typical neurophysiological pattern of a BCI-illiterate user, and a performance predictor combining four different values of spectral band power (theta, alpha, beta, and gamma) was proposed and evaluated using the same data. **Figure 5** demonstrates the comparison between the correlation coefficients of the three methods. Interestingly, the proposed neurophysiological predictor yielded *r* = 0.48 and self-prediction yielded *r* = 0.64. Even though more thorough investigation of physiological, anatomical, and psychological correlates should be conducted using the same dataset, we may conclude from our findings that self-prediction is quite comparable to the existing predictors; moreover, it is an easy and quick way to predict BCI performance because it does not require long preparations for the installation of systems or complex brain imaging.

As we observed, self-prediction may not be the most suitable strategy to quickly determine BCI-illiteracy in advance. However, the result revealed that the domain, which has thus far been overlooked, should be taken into account for investigating BCI-illiteracy. This means that user feedback is just as important as other factors. Thus, it is an interesting topic to introduce user feedback in updating and advancing the current BCI training protocol, control paradigms (e.g., p300 BCI), algorithms, different imaging modality (Functional near-infrared spectroscopy and Magnetoencephalography), and hybrid BCIs.

Here, we discuss the limitations of this study. First the observed self-prediction becomes closer to the session-wise performance that was calculated from the overall trials from 1st run to the last run. Thus, it is possible that the significant correlation could be due either to a good estimation of their performance by the participants or to the fact that subjects become bored and so stop putting effort into performing the task. Considering the statistics of accuracy (mean: 66.9%, median: 63.4%, and standard deviation: 11.0%) and prediction (mean: 63.5%, median: 62.0%, and standard deviation: 8.5%), and the fact that the numbers of subjects whose classification accuracy/or prediction is below than 60% are *N* = 18 (accuracy), *N* = 16 (prediction), and *N* = 9 (both), the latter reason might influence the result. If this is true, it could cause the performance to occur around chance level.

**FIGURE 6:**
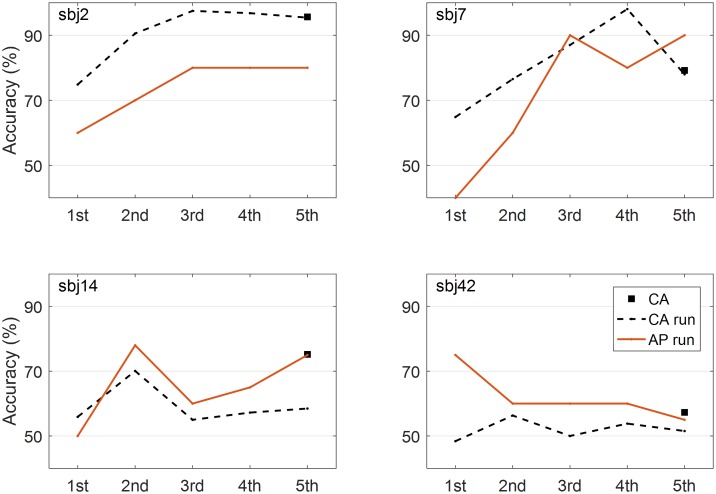
Evolutions of performance and prediction. Four representative figures show how the performances and predictions evolve across runs. Classification accuracy (CA) from all the trials (5 runs), classification accuracy (CA run) at each run, and accuracy prediction (AP) at each run are presented.

To address this issue, the performance at each run was estimated using the same method (CSP with six filters and FLDA), and the evolutions of performance and prediction were investigated for all subjects. **Figure 6** represents the results from the four representative subjects. As a result, it was not the case that performance goes to the chance level and prediction is around chance level, too. However, only 20 trials per class were collected in this study, so obtaining the trustable classification accuracy is difficult when comparing the both accuracy and prediction at each run because the small number of trials dramatically increases the chance level (Müller-Putz et al., 2008). In addition, considering the relatively large portion of low performers (*N* = 16), further investigation with sufficient number of trials should be done to reach a solid conclusion on this issue.

Second, only the task without feedback was evaluated in this study, but considering the report that training with feedback (e.g., neuro-feedback based training (Hwang et al., 2009) helps users to modulate classifiable brain waves in BCI, it is interesting to see how much influence that feedback from the system has on building the sense of motor imagery quality; such feedback may be helpful or may cause users to have a biased model of self-sensing. We believe that direct comparison of two tasks with or without feedback would give a greater indication of the influence of feedback on the internal model, which will be under investigation.

Third, we used the general techniques (i.e., CSP and FLDA) common in the BCI field, and sixteen subjects showed accuracy lesser than 60%, which may be considered a chance level. Therefore, it is worthwhile to check if such population might have an influence on the results. However, re-computation without such subjects showed that the high correlation is still observable (*r* = 0.67) as seen on Supplementary Figure 1. More complex algorithms may find the better features and construct more accurate decision hyperplane, thus yielding higher classification accuracy. But considering the existing literature (Samek et al., 2012; Lotte, 2015; Alimardani et al., 2017), the order of performance across subjects is hardly changed. Therefore, we believe that a higher-order ML algorithm will not weaken the significance of the result.

## Conclusion

We investigated the correlations between the personal information/conditions and self-assessed motor imagery scores of study participants with actual (offline) classification accuracy in MI-BCI. None of the personal information or conditions were statistically significantly correlated with actual classification accuracy. However, we observed a high positive correlation between the self-predicted BCI performance and actual classification accuracy. Additionally, our results demonstrate that such self-prediction improves as the subjects experience more of the motor imagery trials (after roughly 16 min), even though there was no feedback to subjects regarding performance. In conclusion, the introduction of a self-prediction by a BCI user is also useful information for understanding BCI performance variation.

## Ethics Statement

This study was carried out in accordance with the recommendations of the Institutional Review Board in Gwangju Institute of Science and Technology with written informed consent from all subjects. All subjects gave written informed consent in accordance with the Declaration of Helsinki. The protocol was approved by the Institutional Review Board in Gwangju Institute of Science and Technology.

## Author Contributions

MA, HC, SA, and SJ designed the study. MA, HC, and SA collected data. MA analyzed data and wrote the manuscript. MA and SJ contributed to the editing of the final manuscript.

## Conflict of Interest Statement

The authors declare that the research was conducted in the absence of any commercial or financial relationships that could be construed as a potential conflict of interest.
