# Supplementary material for: User’s Self-Prediction of Performance in Motor Imagery Brain–Computer Interface
Source: Front Hum Neurosci. 2018 Feb 15;12:59. doi: 10.3389/fnhum.2018.00059 (PMC5818431; doi:10.3389/fnhum.2018.00059)
Supplement: Supplementary file 1 [file Presentation_1.PDF]

Supplementary Material

User's self-prediction of performance in motor imagery brain-computer interface

Minkyu Ahn, Hohyun Cho, Sangtae Ahn and Sung Chan Jun*

*** Correspondence:** Corresponding Author: scjun@gist.ac.kr

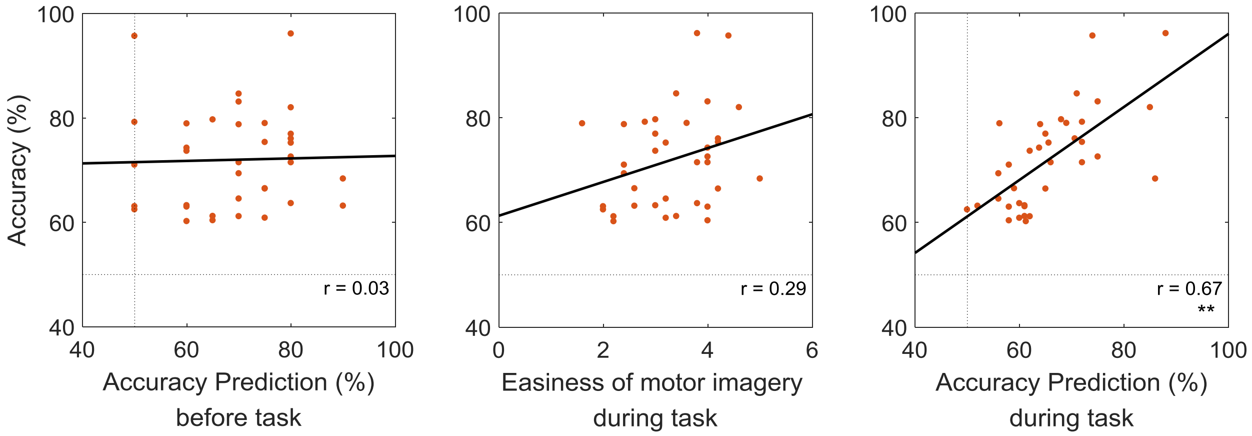

Supplementary Figure 1. Correlations with actual classification accuracy. Only subjects who showed over 60% classification accuracy were used.

Supplementary Table 1. Accuracy prediction (AP) of each run.

ID	1st run AP	2nd run AP	3rd run AP	4th run AP	5th run AP	AP	ACC
sbj1	90	90	85	85	90	88	96.11
sbj2	60	70	80	80	80	74	95.65
sbj3	60	70	80	75	70	71	84.60
sbj4	80	80	70	70	75	75	83.07
sbj5	85	85	85	85	85	85	81.97
sbj6	60	70	70	70	70	68	79.65
sbj7	40	60	90	80	90	72	79.18
sbj8	65	70	70	65	75	69	78.96
sbj9	55	55	60	56	55	56.2	78.89
sbj10	60	60	60	70	70	64	78.72
sbj11	60	65	65	65	70	65	76.90
sbj12	70	70	78	85	50	70.6	76.00
sbj13	60	60	80	85	75	72	75.33
sbj14	50	78	60	65	75	65.6	75.19
sbj15	58	62	67	62	70	63.8	74.24
sbj16	60	60	60	60	70	62	73.64
sbj17	60	65	75	85	90	75	72.54
sbj18	80	70	70	70	70	72	71.44
sbj19	70	65	60	70	65	66	71.44
sbj20	70	65	50	50	55	58	70.99
sbj21	45	65	65	50	55	56	69.33
sbj22	90	90	90	80	80	86	68.33
sbj23	60	60	60	60	55	59	66.47
sbj24	60	70	60	75	60	65	66.42
sbj25	60	55	55	55	55	56	64.50
sbj26	70	60	50	60	60	60	63.63
sbj27	60	65	60	60	60	61	63.22
sbj28	40	50	55	55	60	52	63.14
sbj29	60	60	65	60	60	61	63.04
sbj30	60	55	55	60	60	58	62.96
sbj31	50	50	50	50	50	50	62.43
sbj32	60	60	60	60	65	61	61.17
sbj33	65	55	65	65	60	62	61.13
sbj34	50	70	60	60	60	60	60.83
sbj35	65	60	50	55	60	58	60.35
sbj36	-	60	60	65	60	61.25	60.18
sbj37	60	60	60	50	65	59	59.71
sbj38	60	50	50	50	50	52	59.19
sbj39	60	75	60	50	60	61	58.07
sbj40	50	50	51	50	51	50.4	57.69
sbj41	50	50	60	50	50	52	57.64
sbj42	75	60	60	60	55	62	57.29
sbj43	60	55	60	60	60	59	56.76
sbj44	50	50	60	60	60	56	56.68
sbj45	60	65	65	66	65	64.2	55.81
sbj46	60	60	55	55	60	58	55.40
sbj47	70	65	65	60	60	64	54.57
sbj48	80	70	58	60	62	66	54.11
sbj49	60	60	65	65	60	62	53.16
sbj50	55	65	60	55	55	58	52.58
sbj51	-	55	-	-	-	55	52.14
sbj52	60	-	70	65	70	66.25	47.90
